# *RGG1*, Involved in the Cytokinin Regulatory Pathway, Controls Grain Size in Rice

**DOI:** 10.1186/s12284-020-00436-x

**Published:** 2020-11-10

**Authors:** Yajun Tao, Jun Miao, Jun Wang, Wenqi Li, Yang Xu, Fangquan Wang, Yanjie Jiang, Zhihui Chen, Fangjun Fan, Mengbin Xu, Yong Zhou, Guohua Liang, Jie Yang

**Affiliations:** 1 Institute of Food Crops, Jiangsu Academy of Agricultural Sciences/Nanjing Branch of Chinese National Center for Rice Improvement, Nanjing, 210014 Jiangsu China; 2 Jiangsu Co-Innovation Center for Modern Production Technology of Grain Crops, Yangzhou University, Yangzhou, 225009 Jiangsu China; 3 Jiangsu Key Laboratory of Crop Genetics and Physiology/Key Laboratory of Plant Functional Genomics of the Ministry of Education, Yangzhou University, Yangzhou, 225009 China

**Keywords:** Heterotrimeric G protein, *RGG1*, Rice, Grain size, Cytokinin

## Abstract

Heterotrimeric GTP binding proteins (G proteins) and cytokinin play important roles in regulating plant growth and development. However, little is known about the mechanism by which they coordinate the regulation of grain size in rice. We functionally characterized one gene, *RGG1*, encoding a type-A Gγ subunit. Strong GUS staining was detected in young panicles and spikelets, suggesting a role for this gene in modulating panicle-related trait development. Overexpression of *RGG1* in Nipponbare (NIP) and Wuyunjing 30 (WYJ30) significantly decreased plant height, panicle length and grain length by regulating cell division. However, *rgg1* mutants generated by the CRISPR/Cas9 system exhibited no obvious phenotypic differences, which may be due to the extremely low expression level of this gene in vivo. The transcriptomes of young panicles of NIP, the NIP-*rgg1–2* mutant and the NIP-OE2 overexpression line were sequenced, and the results showed that many differentially expressed genes (DEGs) were associated with the cytokinin biosynthetic pathway. We confirmed this result by measuring the endogenous cytokinin levels and found that cytokinin content was lower in the overexpression lines. Additionally, increased expression of *RGG1* decreased sensitivity to low concentrations of 6-benzylaminopurine (6-BA). Our results reveal a novel G protein—cytokinin module controlling grain size in rice and will be beneficial for understanding the mechanisms by which G proteins regulate grain size and plant development.

**Supplementary Information:**

The online version contains supplementary material available at 10.1186/s12284-020-00436-x.

## Background

Heterotrimeric GTP binding proteins (G proteins) are key regulators of a multitude of transmembrane signalling pathways in animals and plants. The heterotrimeric G protein complex is composed of Gα, Gβ, and Gγ subunits, which cycle between active and inactive forms. G protein signalling is activated by seven-pass transmembrane G protein–coupled receptors (GPCRs) that function as guanine nucleotide exchange factors and then transduce the signal to downstream effectors (Pandey 2019). In plants, G proteins are involved in multiple fundamental growth and development pathways, including panicle branching (Huang et al. 2009; Zhou et al. 2009), seed size (Liu et al. 2018; Mao et al. 2010; Sun et al. 2018), shoot apical meristem (SAM) development (Bommert et al. 2013), nitrogen utilization (Sun et al. 2014), and stress tolerance (Yu and Assmann 2015; Zhang et al. 2015).

Although G proteins are evolutionarily conserved, their numbers vary widely between humans and plants. For example, at least 23 Gα, 5 Gβ and 12 Gγ have been identified in humans. In contrast, the rice genome contains only one Gα (*RGA1*), one Gβ (*RGB1*), and five Gγ homologs (*RGG1*, *RGG2*, *GS3*, *qPE9–1*/*DEP1*, and *GGC2*) (Sun et al. 2018). Mutation in *RGA1* causes severe dwarfing and small grain size (Zong et al. 2018). Additionally, *RGA1* is involved in regulating rice gibberellin and brassinosteroid signalling (Ueguchi-Tanaka et al. 2000; Wang et al. 2006). The Gβ gene *RGB1* positively regulates cellular proliferation to modulate internode elongation and grain size (Utsunomiya et al. 2011). Evidence also shows that *RGB1* functions as a positive regulator of ABA to modulate rice drought tolerance (Zhang et al. 2015). The five Gγ proteins antagonistically regulate grain length in rice. In particular, GS3, qPE9–1/DEP1, and GGC2 competitively interact with Gβ to control grain size (Sun et al. 2018). qPE9–1/DEP1 could directly interact with the MADS-domain transcriptional factor OsMADS1 and enhance its transcriptional activity to modulate grain size (Liu et al. 2018). *RGG2*, encoding a type B Gβ subunit, negatively regulates grain size and is also involved in gibberellin signalling (Miao et al. 2019). These prior studies in rice reveal that G proteins play vital roles in the determination of grain size as well as in phytohormone regulation.

Phytohormones play diverse roles in plant growth and development (Blázquez et al. 2020). Cytokinin, one of the most important phytohormones, has been shown to modulate panicle traits. *Gn1a* encodes a cytokinin oxidase/dehydrogenase enzyme that is responsible for cytokinin degradation in vivo. Mutation in *Gn1a* results in cytokinin accumulation and causes an increase in grain number per panicle (GN) (Ashikari et al. 2005). Conversely, the cytokinin-activating enzyme LONELY GUY (LOG) directly converts inactive cytokinin to biologically active forms. The *log* mutant has highly reduced SAMs and panicles and abnormal branching patterns (Kurakawa et al. 2007). Cytokinin signalling plays important roles in regulating meristem cell proliferation and differentiation (Stahl and Simon 2010). Studies have shown that G proteins are also involved in stem cell fate determination. Maize *COMPACT PLANT2* (*CT2*), which encodes a Gα subunit, interacts with FASCIATE EAR2 (CLV2) to regulate inflorescence meristem size (Bommert et al. 2013). In *Arabidopsis*, Gβ mutants showed an enlarged meristem size (Ishida et al. 2014). AGB1 interacts with RPK2, one of the CLV3 peptide hormone receptors, to regulate meristem development. However, rice G proteins involved in CLAVATA signalling have not been reported.

Decreased levels of cytokinin also lead to reduced grain size in the *root enhancer1* (*ren1-D*) mutant due to activation of *OsCKX4* (Gao et al. 2014), and grain size may be regulated in part by modulation of long-distance transport of cytokinin by *Big Grain3* (*BG3*), which encodes a purine permease, *OsPUP4* (Xiao et al. 2019). Recently, the Gγ subunit *qPE9–1*/*DEP1* was found to positively regulate grain filling by increasing endogenous cytokinin and auxin concentrations in rice grains (Zhang et al. 2019). However, how G proteins interact with cytokinin signalling to control growth and development in plants remains largely unknown.

In this study, we functionally analysed the γ-subunit gene *RGG1* in rice. Overexpression of *RGG1* caused reduced plant height and grain length. Further results suggested that *RGG1* modulates endogenous cytokinin accumulation and responses to regulate plant morphology and grain development.

## Results

### *RGG1* Encodes a Type-A Gγ Subunit

**Fig. 1:**
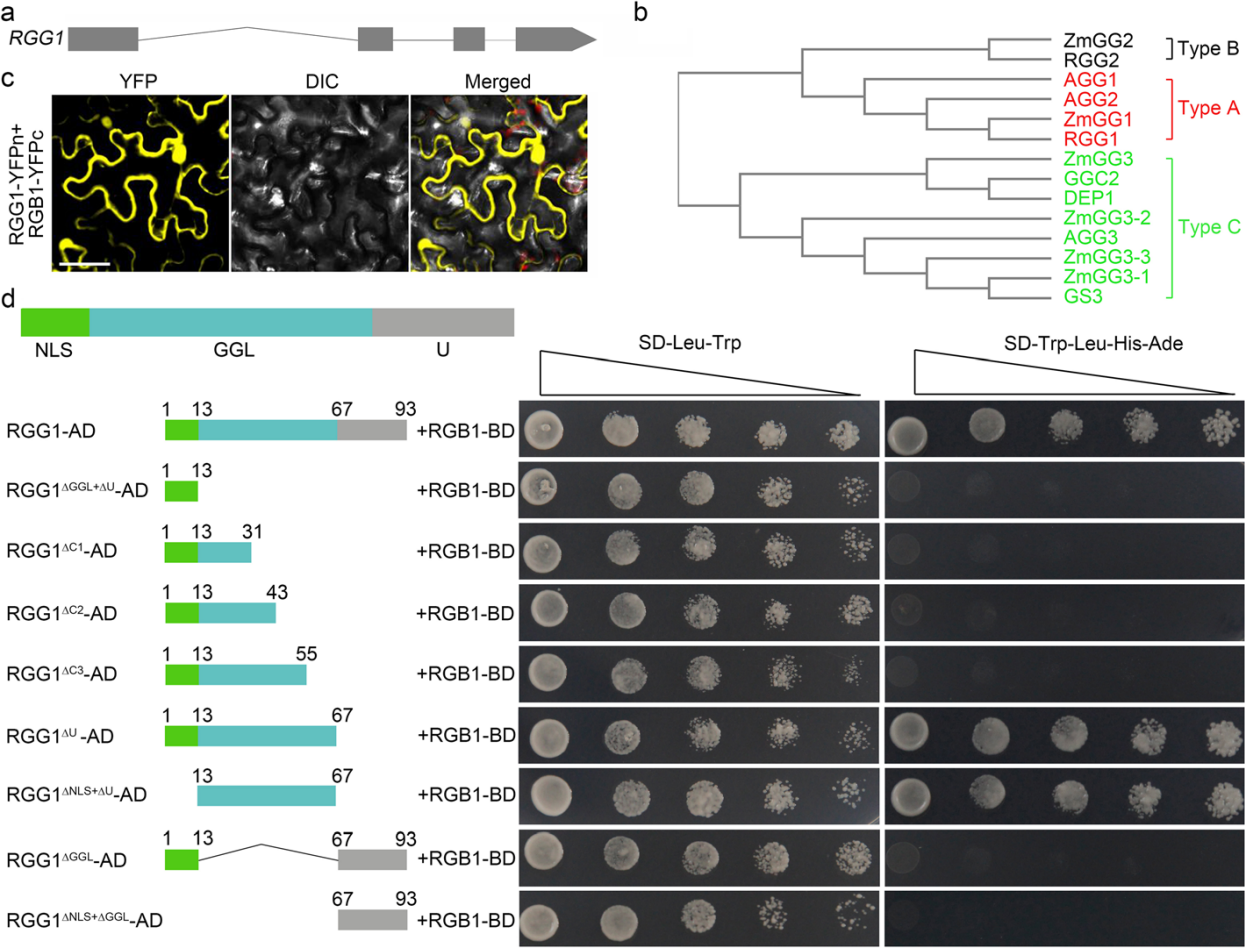
RGG1 encodes a type-A Gγ subunit. **a** Gene structure of *RGG1*. **b** Phylogenetic tree of Gγ subunits from rice, Arabidopsis and maize. **c** Interaction between RGG1 and RGB1. Scale bars, 100 μm. **d** Yeast two-hybrid assay. In this assay, RGG1 was used as the prey (GAL4-AD, AD) due to its autoactivation ability, and RGB1 was used as the bait (GAL4-BD, BD). Δ represents deleted protein parts. The numbers show the different lengths of each truncated protein. NLS is the predicted nuclear localization signal. GGL is the G gamma-like domain. U represents the unknown domain

In the rice genome, five Gγ subunits have been identified. Among them, RGG1 is relatively small and contains four exons (Fig. 1a). Phylogenetic analysis showed that the G proteins of rice, *Arabidopsis*, and maize were divided into three groups (Fig. 1b). Among types A, B and C, the amino acid sequences showed very little conservation, and most of the similarities were limited to a highly conserved GGL (G gamma-like) domain (Fig. S1). *RGG1* belongs to a clade of type-A G proteins along with the *AGG1* and *AGG2* proteins of *Arabidopsis*. SMART analysis predicted that *RGG1* contains a nuclear location signal (NLS) at the N-terminus, a GGL domain, and a CaaX isoprenylation motif at the C-terminal end, typical of all canonical type-A G proteins (Fig. S1).

We confirmed that RGG1 interacts with RGB1 using a bimolecular fluorescent complementation (BiFC) assay. The BiFC fluorescence signal was detected in the membrane, cytoplasm and nucleus, suggesting the potential function of the Gβγ dimer (Fig. 1c). To further explore the interaction of RGG1 and RGB1, several truncated RGG1 proteins were generated. As shown in Fig. 1d, the GGL domain was necessary and sufficient for interaction with RGB1. In addition, residues 55–67 of RGG1 were required for the RGG1-RGB1 interaction (Fig. 1d).

### Expression Profiles and Subcellular Localization

**Fig. 2:**
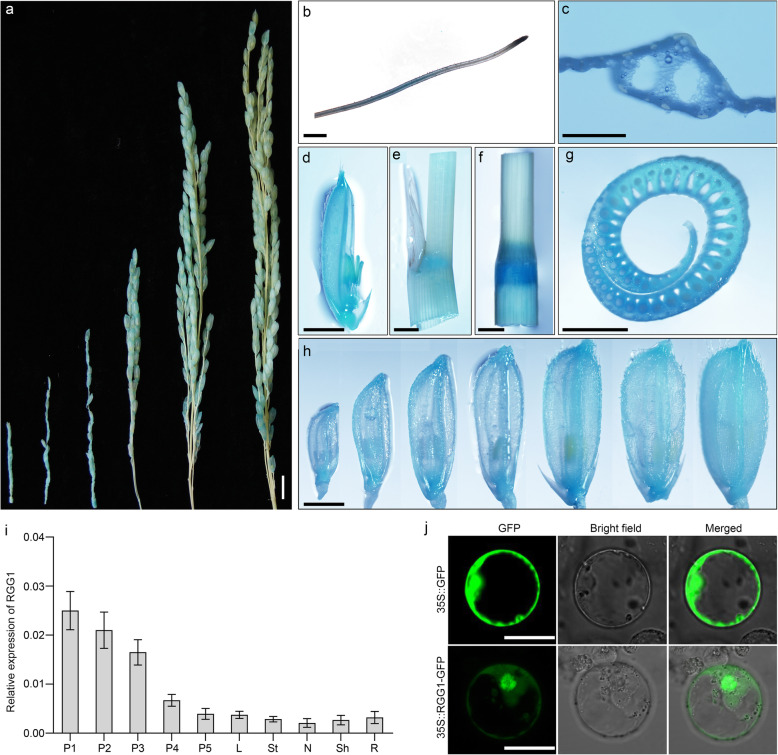
Molecular characterization of RGG1. **a** GUS activity in young panicles at different developmental stages. Scale bar, 1 cm. **b** GUS activity in roots. Scale bar, 1 mm. **c** GUS activity in leaves. Scale bar, 50 μm. **d** GUS activity in spikelets. Scale bar, 2 mm. **e** GUS activity in sheaths. Scale bar, 2 mm. **f** GUS activity in stem nodes. Scale bar, 2 mm. **g** GUS activity in node cross-sections. Scale bar, 2 mm. **h** GUS activity in spikelets during different developmental stages. Scale bar, 2 mm. **i** *RGG1* transcript levels in different tissues. P1-P5, young panicles with average lengths of approximately 3 cm, 6 cm, 10 cm, 13 cm and > 15 cm, respectively. L, leaf. St, stem. N, node. Sh, sheath. R, root. **j** Subcellular localization of RGG1 in rice protoplasts. Scale bar, 20 μm

To determine the expression pattern of RGG1, the tissue-specific expression of *RGG1* was detected using transgenic plants containing an *RGG1* promoter: GUS fusion. GUS staining revealed different levels of expression of *RGG1* in panicles at different developmental stages. As shown in Fig. 2a, the expression of *RGG1* gradually decreased with panicle development. It was also expressed in roots, with particularly strong staining in the root tips (Fig. 2b). Additionally, GUS staining showed that *RGG1* was abundantly expressed in leaves, sheaths, nodes, stems, and spikelets (Fig. 2c-h). Moreover, the GUS results were in agreement with our quantitative reverse transcription-PCR (qPCR) analyses, which showed particularly high expression of *RGG1* in young panicles and decreasing panicle expression as development progressed (Fig. 2i). In addition, we detected *RGG1* transcripts in other tissues using qPCR, including leaves, stems, nodes, sheaths, and roots (Fig. 2i). These data suggest that *RGG1* may play an important role in panicle and seed development.

To observe the subcellular localization of RGG1, both green fluorescent protein (GFP) and an RGG1-GFP fusion protein driven by the CaMV 35S promoter were transiently expressed in rice protoplasts. Similar to the GFP signal, RGG1-GFP was detected in the plasma membrane, cytoplasm and nucleus (Fig. 2j). To verify the function of the predicted NLS at the N-terminus, we also transiently expressed a truncated protein, RGG1_ΔNLS_-GFP, in rice protoplasts. However, the fluorescent signal of RGG1_ΔNLS_-GFP showed the same distribution as that of RGG1-GFP, suggesting that the putative NLS domain may not be functional (Fig. S2).

### Overexpression of *RGG1* Resulted in Yield Reduction in Nipponbare Rice

**Fig. 3:**
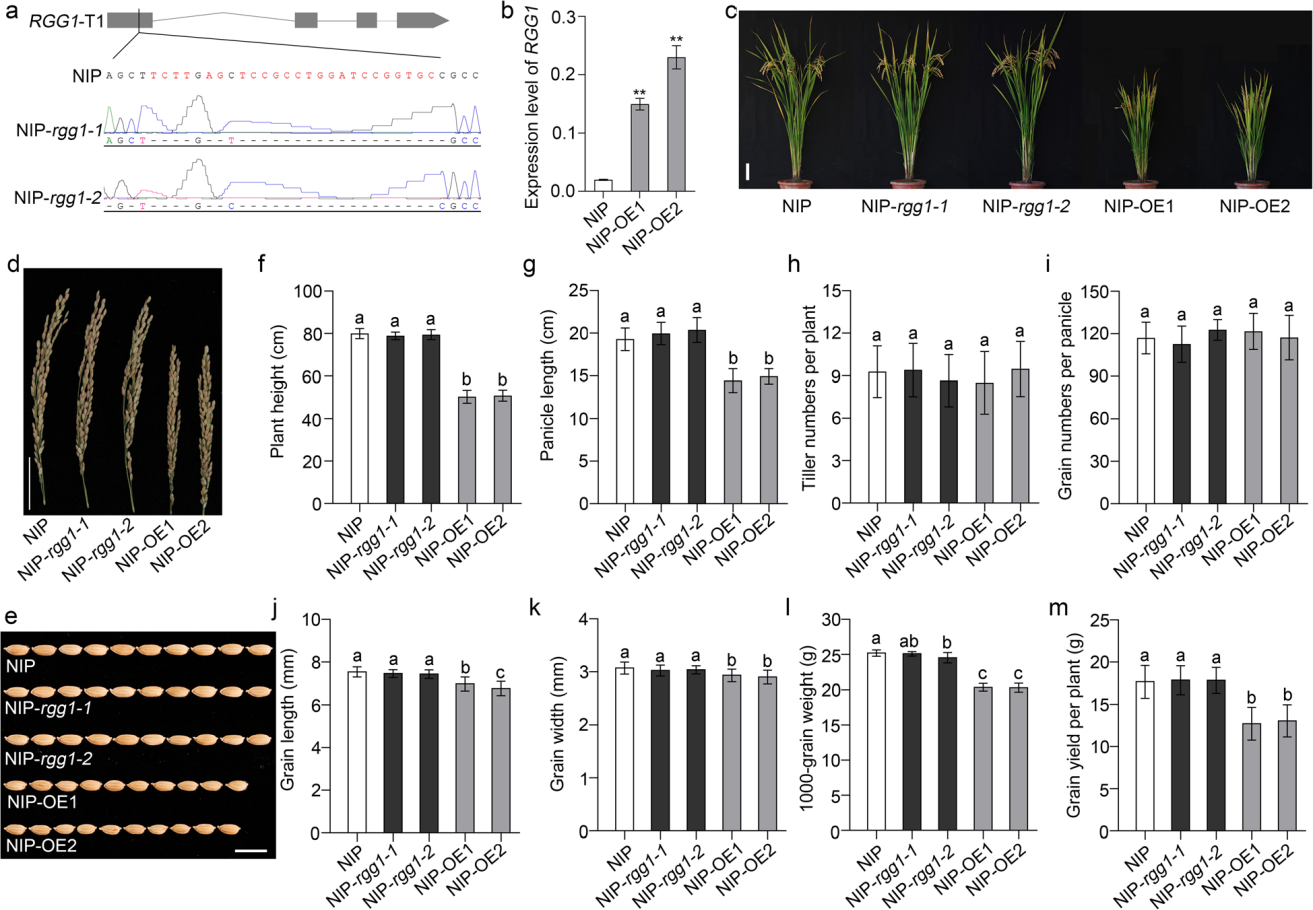
Overexpression of *RGG1* has multiple effects on agronomic traits. **a** Targeted mutation of *RGG1* using the CRISPR/Cas9 system generated two mutants (NIP-*rgg1–1* and NIP-*rgg1–2*), which were confirmed by sequencing. **b** Relative expression levels of NIP and two RGG1 overexpression lines (NIP-OE1 and NIP-OE2). *OsActin* was selected as the internal control. **c** Plant morphology of NIP, NIP-*rgg1–1*, NIP-*rgg1–2*, NIP-OE1 and NIP-OE2 at the mature stage. Scale bar, 10 cm. **d** Panicle phenotypes of NIP, NIP-*rgg1–1*, NIP-*rgg1–2*, NIP-OE1 and NIP-OE2. Scale bar, 5 cm. **e** Grain sizes of NIP, NIP-*rgg1–1*, NIP-*rgg1–2*, NIP-OE1 and NIP-OE2. Scale bar, 1 cm. **f**-**m** Comparisons among NIP, the mutants and the OE lines with respect to **f** plant height; **g** panicle length; **h** tiller number per plant; **i** grain number per panicle; **j** grain length; **k** grain width; **l** 1000-grain weight; and **m** grain yield per plant. The data are given as the mean ± SD (*n* ≥ 20). Different letters indicate significant differences ranked by the LSD test (*P* < 0.05)

To elucidate the biological function of *RGG1*, overexpression and knockout vectors were generated and then transformed into NIP using an *Agrobacterium tumefaciens*-mediated method. Several successful transformed lines were obtained and confirmed by qPCR and sequencing. We chose two overexpression (OE) and two mutant lines for further analysis (Fig. 3a, b).

The relative expression levels of *RGG1* in two OE lines (OE1 and OE2) were detected. Compared to that in NIP, the expression level of *RGG1* was higher by eight- and twelve-fold in OE1 and OE2, respectively (Fig. 3b). As a result, the OE1 and OE2 transgenic lines showed a semi-dwarf phenotype at maturity (Fig. 3c). Further analysis showed that all the internode lengths of the OE lines were shorter than those of NIP (Fig. S3a, b). Additionally, we quantified other yield components, such as panicle length (PL), tiller number per plant (TN), GN and 1000-grain weight (TGW) (Fig. 3h, i, l, Table S1). Neither PN nor TN showed a difference between NIP and the two OE lines (Fig. 3h, i). However, the TGW values of OE1 and OE2 decreased by 19.20% and 19.44%, respectively, compared to that of NIP (Fig. 3l). Further analysis suggested that *RGG1* affects grain length and width but has no influence on grain thickness (Fig. 3e-k, Table S1). In particular, the grain lengths in the OE lines were lower by 7.41% and 10.17%, respectively, than that in NIP (Fig. 3j). As expected, OE1 and OE2 also exhibited decreased grain yield per plant (Fig. 3m). Taken together, these results indicate that overexpression of *RGG1* can cause semi-dwarf height and shortened grain length.

Additionally, two knockout mutants of *RGG1* were generated using the CRISPR/Cas9 system in the NIP background (Fig. 3a). Sequencing results showed that both mutants, NIP-*rgg1–1* and NIP-*rgg1–2,* had large deletions in the target site that abolished protein expression. However, the mutant plants of lines NIP-*rgg1–1* and NIP-*rgg1–2* did not show any obvious phenotype in traits including plant height and other yield component (Fig. 3c-m). This result may be due to the extremely low expression level of *RGG1* in NIP (Fig. 3b). Whether the role of *RGG1* in signal transduction is subject to genetic redundancy needs further study.

### Overexpression of *RGG1* in Wunyunjing 30 Results in a Similar Phenotype

To investigate whether *RGG1* shows similar effects to those in the NIP mutants in the *qpe9–1/dep1* mutant background, we transformed Wunyunjing 30 (WYJ30), a high-yield variety of rice that naturally lacks a functional *qpe9–1/dep1*, with the *RGG1* overexpression vector, and we measured plant height and other yield-related traits at maturity. Both the WYJ30-OE1 and WYJ30-OE2 lines showed reduced plant height and PL compared to those of WT-WYJ30 (Fig. S4a-d, Table S2). Additionally, compared to WT-WYJ30, the grain lengths of the two OE lines were reduced by 3.29% and 3.42%, respectively (Fig. S4e). There was no significant difference in grain width between the WYJ30 and OE lines (Fig. S4f). In contrast, overexpressing *RGG1* caused decreased TGW and grain yield in WYJ30 (Fig. S4g, h). These results suggest that the roles of *RGG1* in regulating plant height and grain length are independent of *qPE9–1/DEP1*. Pyramiding different Gγ-encoding genes may be a suitable way to modulate grain size in rice.

We also used the CRISPR/Cas9 method in WYJ30 to obtain several homozygous mutants of *RGG1*. We identified one line, WYJ30*-rgg1–1*, with a 4-bp deletion and another with a 1-bp insertion, WYJ30*-rgg1–2* (Fig. S5). Both mutations disrupted the GGL domain (Fig. S5). We observed no changes in plant morphology or grain size between WT-WYJ30 and these two mutants (Table S2). Taken together, knockout of *RGG1* might not affect rice growth and development.

### RGG1 Regulates Grain Size by Affecting Cell Division

**Fig. 4:**
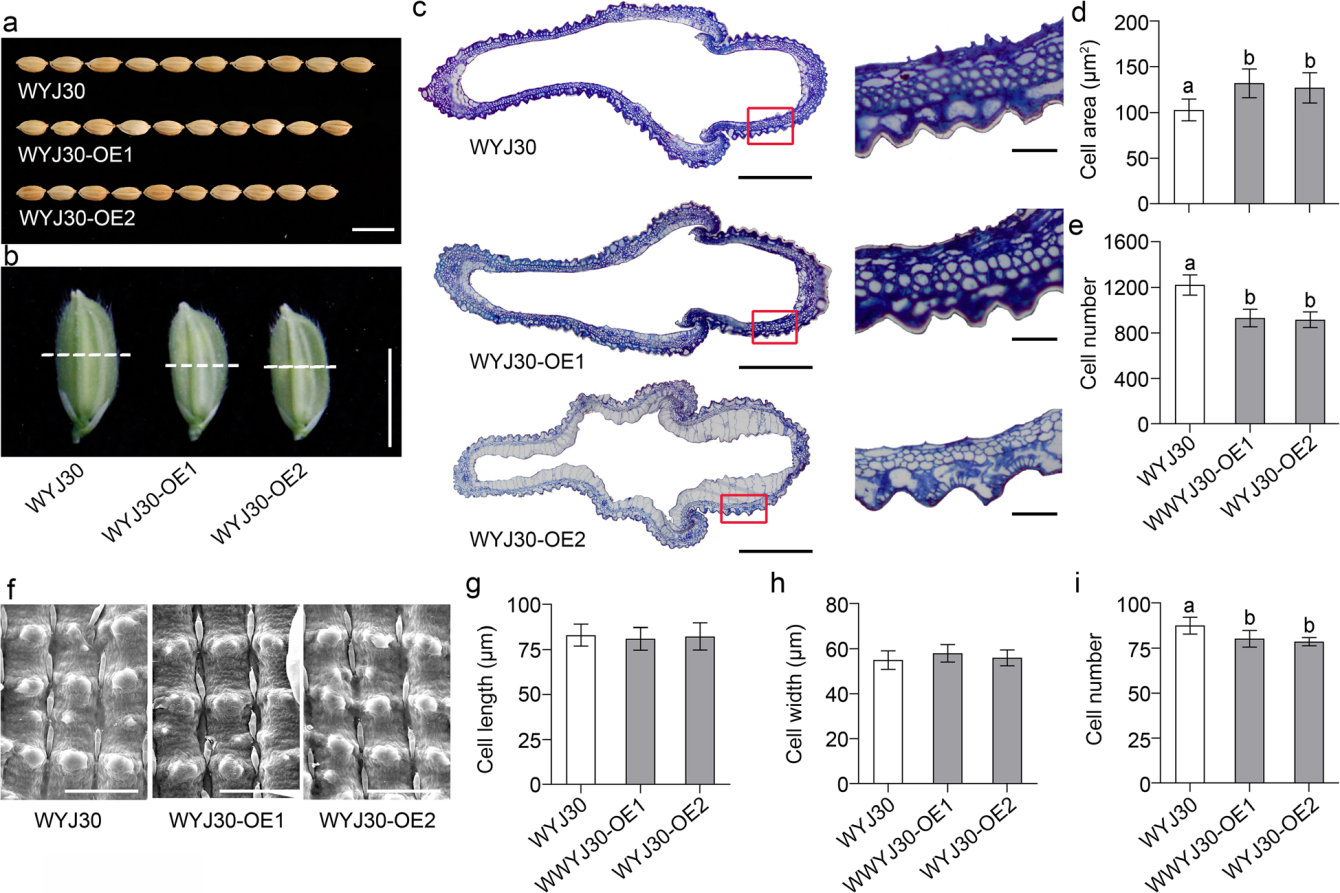
Histological comparison of spikelet hulls between WYJ30 and two OE lines. **a** Grain sizes of WYJ30, WYJ30-OE1 and WYJ30-OE2. Scale bar, 1 cm. **b** Spikelet hulls of WYJ30, WYJ30-OE1 and WYJ30-OE2. Scale bar, 5 mm. **c** Cross-sections of the middle parts of the spikelet hulls (marked by white dashed lines in **b**) of WYJ30 and the OE lines. Scale bar, 500 μm. Magnified views of the red boxed areas are shown on the right. Scale bar, 50 μm. **d** Cell area and **e** cell number in the outer parenchyma layer of the spikelet hulls of WYJ30 and the OE lines. **f** Scanning electron micrographs of the lemma cells of WYJ30 and the OE lines. Scale bar, 100 μm. **g** Cell lengths, **h** cell widths and **i** longitudinal cell numbers of the WYJ30 and OE lines. The data are given as the mean ± SD (*n* ≥ 15). Different letters indicate significant differences ranked by the LSD test (*P* < 0.05)

The spikelet hull has an important impact on grain size determination. Compared with WYJ30, both OE lines had reduced grain lengths and grain widths (Fig. 4a, b). Generally, organ size is determined by cell expansion and division. To investigate the grain size differences between the WYJ30 and OE lines, histological cross-sections of the spikelet hulls were analysed (Fig. 4c-e). As shown in Fig. 4d and e, both the OE lines had significantly higher cell areas and lower cell numbers than WYJ30. Furthermore, the epidermal cells of WYJ30 and the transgenic lines were analysed using scanning electron microscopy (SEM) (Fig. 4f). No obvious difference in cell length or cell width was found between the WYJ30 and OE lines (Fig. 4g, h). However, the OE lines had fewer longitudinal cells than WYJ30 (Fig. 4i). Overall, these results suggest that overexpressing *RGG1* suppressed cell division in the spikelet hull and consequently led to smaller grain size.

### *RGG1* is Involved in Cytokinin Biosynthesis

**Fig. 5:**
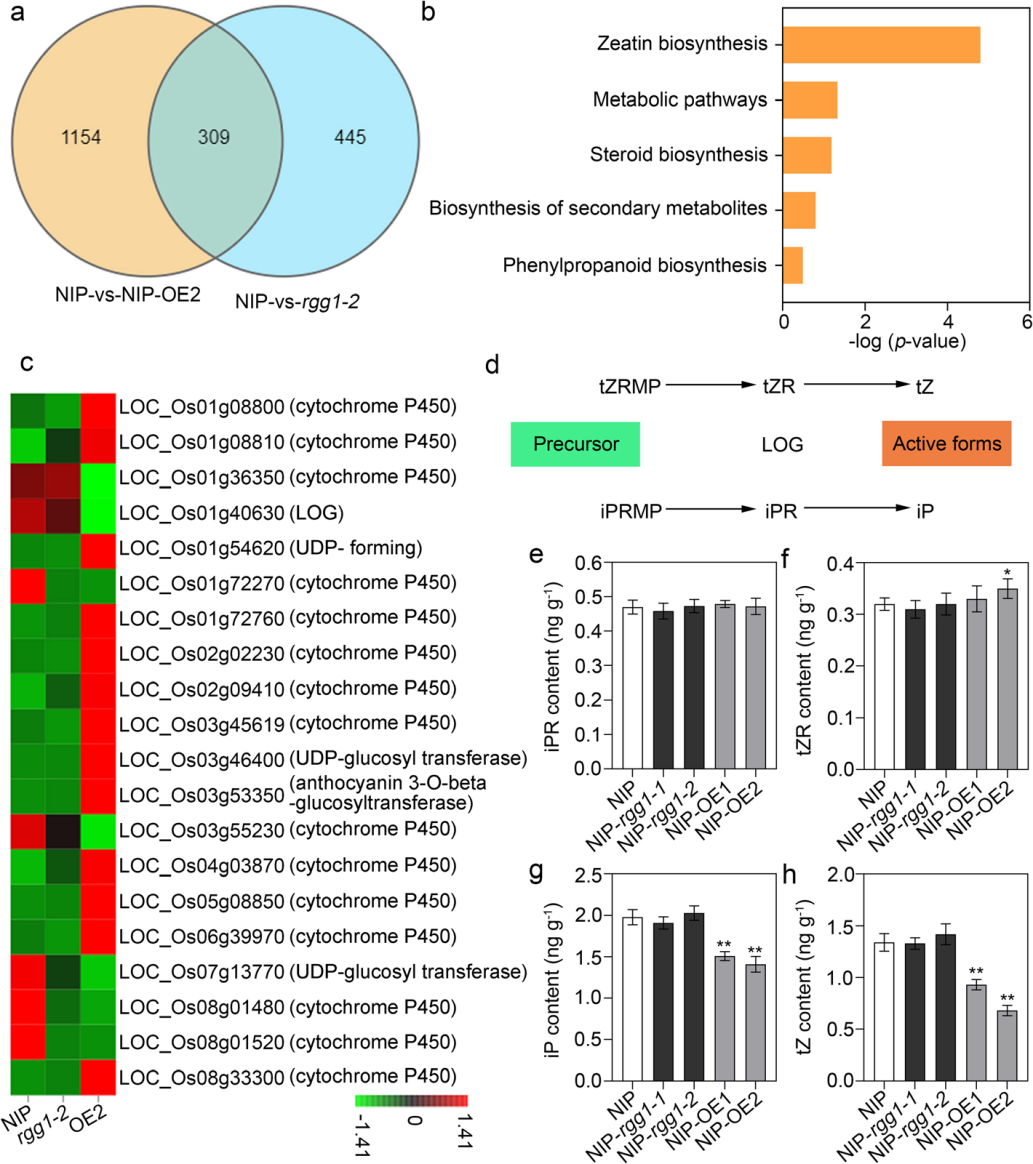
Transcriptome profiles of NIP, *rgg1–2* and NIP-OE2. **a** Differentially expressed genes (DEGs) in the young panicles of NIP and the transgenic lines. The young panicles of NIP, *rgg1–2* and NIP-OE2, including 3 biological replicates, were collected for analysis. **b** Kyoto Encyclopedia of Genes and Genomes (KEGG) enrichment of DEGs. **c** Heat map of DEGs associated with cytokinin biosynthetic and regulatory pathways. The different colours in each box indicate the Z-score values. **d** Schematic of the cytokinin biosynthetic pathway from precursors (tZRMP and iPRMP) to active forms (tZ and iP). tZRMP, trans-zeatin riboside-5′-monophosphate. iPRMP, N6-(Δ2-isopentenyl) adenine riboside monophosphate. tZ, trans-zeatin. iP, isopentenyladenine. **e** iPR content, **f** tZR content, **g** iP content and **h** tZ content in young panicles of NIP and the transgenic lines (with three biological replicates). iPR, N6-(Δ2-isopentenyl)adenosine. tZR, trans-zeatin-riboside. The data are given as the mean ± SD (*n* = 3). Student’s *t*-test: *P < 0.05, **P < 0.01

Due to the significant influences of RGG1 on panicle elongation and grain length that we observed, we then performed a transcriptome analysis to investigate the possible molecular pathway of *RGG1* action in the young panicles of NIP, NIP-*rgg1*–*2*, and NIP-OE2. A total of 1463 differentially expressed genes (DEGs) were detected in OE2 compared with NIP; 690 of these genes were upregulated, and 773 genes were downregulated (Fig. 5a). Additionally, 754 DEGs, including 249 up- and 505 downregulated genes, were found in the young panicles of the *rgg1–2* mutant. The detected DEGs were involved in diverse biological processes and metabolic pathways (Fig. S6). Analysis of the DEGs using Gene Ontology showed that the greatest enrichment was in the biological process category. Additionally, Kyoto Encyclopedia of Genes and Genomes (KEGG) analyses revealed that the zeatin biosynthetic pathway was enriched in DEGs (Fig. 5b). In particular, many DEGs were associated with cytokinin biosynthesis (Fig. 5c). Notably, one gene, LOC_Os01g40630, encoding the cytokinin-activating enzyme LOG, which is responsible for converting inactive cytokinin to biologically active forms, was downregulated in the young panicles of NIP-OE2 (Fig. 5c, d). The expression levels of several cytokinin biosynthetic genes were confirmed using qPCR assays (Fig. S7a-c), and LOG was found to be downregulated in the young panicles of the OE lines and upregulated in *rgg1* mutants. We also analysed two other cytokinin biosynthetic genes. CYP735A4, encoding the key enzyme converting isopentenyladenine (iP)-type to trans-zeatin (tZ)-type cytokinins, had decreased expression in the panicles of the OE lines, while no significant changes were observed in the mutants. OsIPT9 encodes IPP transferase for synthesizing cZ in rice (Tsai et al. 2012) and was downregulated in the young panicles of the OE lines and upregulated in the *rgg1* mutants. These results suggest that *RGG1* might be involved in the cytokinin regulatory pathway.

To test this hypothesis, we measured the concentrations of cytokinin in young panicles (Fig. 5e-h). The total contents of two cytokinin precursors, N6-(Δ2-isopentenyl) adenosine (iPR) and trans-zeatin-riboside (tZR), in the OE lines were similar to those in NIP (Fig. 5e, f). However, the contents of the active forms, iP and tZ, were significantly lower in the OE lines than in NIP (Fig. 5g, h). NIP-OE2 accumulated more tZR than NIP did, and this effect may be due to an inefficient conversion ability (Fig. 5f). These results suggest that overexpression of *RGG1* reduced the efficiency of the conversion of cytokinin precursors to active forms, possibly as a result of lower expression of *LOG* or other genes in the cytokinin pathway.

### RGG1 Affects Cytokinin Signalling

**Fig. 6:**
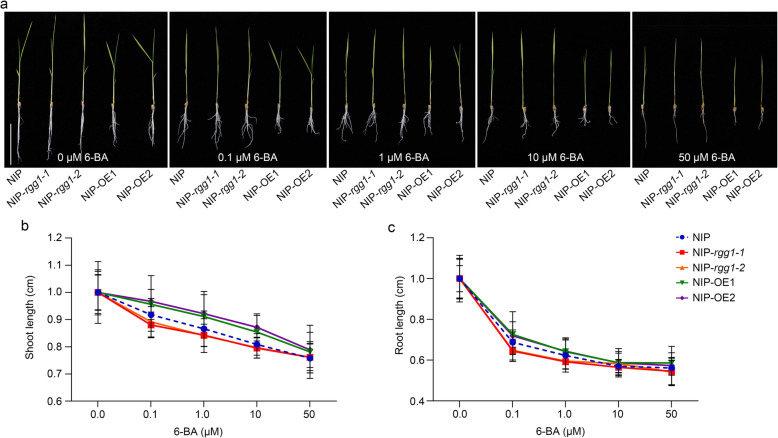
*RGG1* is involved in cytokinin response. **a** Effects of treatment with different concentrations of 6-BA on the growth of NIP and the transgenic lines. Scale bar, 5 cm. **b** Shoot and **c** root responses to 6-BA treatment of NIP and the transgenic lines (*n* ≥ 12)

Heterotrimeric GTP binding proteins (G proteins) are involved in multiple signal transduction processes and intracellular responses to stimuli in plants. We also investigated whether *RGG1* affects cytokinin signal transduction in rice. Shoot and root elongation assays were conducted to test the sensitivity of the overexpression and mutant lines to different concentrations of 6-benzylaminopurine (6-BA) (Fig. 6a). These experiments revealed an altered growth curve for the *RGG1* overexpression lines when treated with 6-BA. At low concentrations, the shoot elongation of NIP and the two mutants was more strongly inhibited than that of OE1 and OE2 (Fig. 6b). The inhibition of root elongation by cytokinin was also compared between NIP and transgenic lines (Fig. 6c). These results showed that the two OE lines had reduced sensitivity to 6-BA with respect to its inhibitory effect on root elongation (Fig. 6c). All these results indicated that *RGG1* is involved in cytokinin biosynthesis and signal transduction in rice.

## Discussion

Grain size is one of the important agronomic traits affecting rice yield and quality. Although a complex regulatory gene network related to grain size has been proposed (Miao et al. 2019; Li and Li 2016), the crosstalk between heterotrimeric G proteins and the cytokinin regulatory pathway in grain size control is poorly understood. In this study, we show that overexpression of *RGG1* resulted in decreased plant height and grain length (Fig. 2 and Fig. S4). Physiological measurements suggested that the active cytokinin level was lower in OE lines than in NIP (Fig. 5), and a 6-BA treatment assay showed that *RGG1* was involved in cytokinin signal transduction. Thus, our findings demonstrate that *RGG1* is involved in cytokinin biosynthesis and signalling and thus controls grain size as well as plant height.

Phylogenetic analysis revealed that rice RGG1 is a type-A Gγ protein. It contains a conserved GGL domain and a CaaX motif at the C-terminus that is characteristic of Gγ subunits among plants and animals and ensures proper membrane targeting (Pandey 2019). Therefore, RGG1 represents a canonical Gγ protein. Subcellular localization results showed that RGG1 localizes to the plasma membrane, cytoplasm, and nucleus (Fig. 2j). Generally, Gα and Gβγ dimers perceive a stimulus at the surface of the cell when in contact with the membrane and then separate to transmit the signal to downstream effectors (Hildebrandt et al. 1984). Previous studies have shown that RGB1, RGG1 and RGG2 localize to the plasma membrane (Kato et al. 2004). However, it is difficult to understand the roles of G proteins within the nucleus. In the nuclei of mammalian cells, Gβγ heterodimers interact with a transcription factor, AP-1, and thus are likely have a transcriptional regulatory role there (Chang et al. 2013; Robitaille et al. 2010). In rice, GS3-GFP and DEP1-GFP are detectable in the membrane and nucleus and function as cofactors of OsMADS1 in regulating grain size (Liu et al. 2018). More recently, the type B Gβ subunit RGG2 was also found to be localized to the plasma membrane, cytoplasm and nucleus (Miao et al. 2019). All of these results suggest that localization of G proteins, including RGG1, to the nucleus is associated with G protein signalling and functions in transcriptional regulation in rice.

Our qPCR and GUS staining results showed that *RGG1* was constitutively expressed in diverse tissues, especially in panicles and spikelets (Fig. 2a-i). To investigate the roles that *RGG1* plays in panicle development, we successfully generated *RGG1* knockout and overexpression lines. The knockout lines *rgg1–1* and *rgg1–2* had no obviously different phenotypes when compared to NIP (Fig. 3, Tables S1 and S2). One possible reason is that *RGG1* normally has very low expression levels in rice (Fig. 3b). In *Arabidopsis*, the *agg1* and *agg2* mutants and *agg1agg2* double mutant exhibit no changes in rosette size, while triple *agg1agg2agg3* mutants show a reduction in rosette size (Thung et al. 2012). Therefore, another possible explanation is that *RGG1* has a redundant function with respect to signal transduction. In contrast, the overexpression lines OE1 and OE2 showed decreased plant height and PL as well as small grains, all of which are consistent with results from a previous study (Liu et al. 2018). In addition, Swain et al. reported that increased *RGG1* expression resulted in increased plant height and enhanced tolerance to salinity stress (Swain et al. 2017). Therefore, we evaluated salinity stress tolerance using our transgenic lines in the NIP background. However, seedlings from the OE lines did not show increased tolerance to salinity stress under treatment with 200 mM NaCl (Fig. S8). Thus, our results support yield reduction and reduced plant height when *RGG1* is overexpressed but not increased height or salt tolerance.

We also overexpressed *RGG1* in the WYJ30 background, which contains a loss-of-function allele of *qpe9–1/dep1*. Similar phenotypes, including a semi-dwarf plant architecture, shortened PL, decreased grain length, and overall lower yield, were also observed in the OE lines (Fig. S4, Table S2). Therefore, *RGG1* and *qPE9–1/DEP1* may function differently in controlling grain size and grain yield characteristics, especially given that WYJ30 is a high-yield variety. Although the roles of Gβ and Gγ subunits in regulating grain size are well known, the mechanisms by which G proteins mediate this process remain poorly understood. For example, Sun et al. crossed GS3-1Ri with RGB1Ri transgenic plants and found that the RGB1Ri/GS3-1Ri hybrid showed reduced grain length. This finding suggests that the effects of grain length increase by GS3-1Ri were dependent on RGB1. Similar results were also obtained in the RGB1Ri/DEP1OE and RGB1Ri/GGC2OE plants. Therefore, *GS3* may have no effect on regulating grain size by itself, while DEP1 and GGC2 compete with RGB1 to modulate grain size. Additionally, a previous study showed that G protein β and γ subunits could physically interact with the transcription factor OsMADS1 to promote its transcriptional activity, thereby controlling grain morphology (Liu et al. 2018). Recently, we functionally analysed one gene, *RGG2*, and found that *RGG2* negatively regulates grain size via the gibberellin pathway (Miao et al. 2019). These prior studies and our present results suggest that the mechanism of regulation of grain size by G proteins is likely very complicated. However, a more complete understanding of how G proteins operate to control grain size in rice is urgently needed to better manipulate rice grain size to meet global consumer demands.

To understand the possible regulatory pathway that *RGG1* mediates, we performed a transcriptome analysis using young panicles from NIP and transgenic plants. The results showed that many DEGs were associated with cytokinin biosynthesis (Fig. 5). In particular, one gene, *LOG*, showed significantly lower expression in the OE lines (Fig. 5c and Fig. S7a). *LOG* is responsible for converting cytokinin precursors to bioactive forms, and its mutant has a defect in inflorescence meristem development (Kurakawa et al. 2007). Cytokinins are adenine derivatives that play essential roles in regulating shoot meristem development (Hwang et al. 2012). However, the relationship between cytokinins and G proteins in mediating developmental processes in plants is poorly understood. In *Arabidopsis*, in addition to the canonical Gα protein, there are three EXTRA-LARGE Gα-like PROTEINs (XLGs) that interact with U-box, PUB, E3 ligases, PUB2 and PUB4, and both the triple mutant *xlgs* and the double mutant *pub2/4* showed defects in cytokinin response. Within the mutant lines, overexpression of *ARR10*, a positive cytokinin response regulator, partially rescued the defective phenotypes (Wang et al. 2017). Recently, Zhang et al. reported that *qPE9–1*/*DEP1* positively regulates grain filling by increasing auxin and cytokinin content in rice grains. Here, we revealed that the concentration of endogenous cytokinin was decreased in the OE lines compared with that in NIP due to the decreased expression of genes encoding cytokinin biosynthetic enzymes. Cytokinin signalling comprises a classic two-step phosphorelay system where an initial signal is transferred to a response regulator (Argueso et al. 2010; Hwang et al. 2012). Here, we also found that *RGG1* is involved in cytokinin signal transduction based on a 6-BA treatment assay (Fig. 6a-c).

Overall, our findings, along with prior work, help to show the complicated crosstalk between G proteins and cytokinin. The critical nature of G proteins in plant development is highlighted by recent reports of knockouts of Gβ (*RGB1*) or Gγ (*RGG2*) in rice or Gβ (*ZmGB1*) in maize causing lethal phenotypes (Gao et al. 2019; Miao et al. 2019; Wu et al. 2020). This may be unsurprising given that G proteins probably mediate shoot meristem size through interaction with CLAVATA receptors (Bommert et al. 2013; Ishida et al. 2014; Wu et al. 2020) and may impact embryo formation via a network between G proteins and cytokinin. We expect that further elucidating the interplay between cytokinin and G proteins will be beneficial for crop improvement via genetic engineering and molecular breeding.

## Conclusions

Altogether, our results showed that overexpression of *RGG1* significantly decreased plant height, panicle length and grain length by regulating cell division in rice. Furthermore, our findings suggested that *RGG1* is involved in cytokinin biosynthesis and signalling pathway. Thus, this study reveals a novel G protein—cytokinin module controlling grain size in rice and will be beneficial for understanding the mechanisms by which G proteins regulate grain size and plant development.

## Materials and Methods

### Plant Materials and Growth Conditions

Both wild-type cultivars (NIP and WYJ30) and transgenic lines of the T_3_ generation were used for phenotypic analyses. These materials were grown on the experimental farm of Yangzhou University following normal agricultural practices. For analyses at the seedling stage, plants were grown in hydroponic culture in a growth chamber with a 12-h light (30 °C) and 12-h dark (28 °C) photoperiod and 70% humidity.

### Homologous Detection and Phylogenetic Analysis

The sequences of the rice, *Arabidopsis* and maize Gγ proteins were obtained from NCBI (https://www.ncbi.nlm.nih.gov/). Multiple alignments were performed using Clustal X. Maximum likelihood (ML) and neighbour-joining (NJ) methods were adopted for the phylogenetic analysis using MEGA v7.0. The ML phylogenetic analyses were conducted with the following parameters: Jones-Taylor-Thornton (JTT) model, estimated proportion of invariable sites, 4 rate categories, estimated gamma distribution parameter, and optimized starting BIONJ tree. In addition, the JTT model was employed for the construction of NJ trees. A total of 1000 non-parametric bootstrap samplings were carried out to estimate the support level for each internal branch for both the ML and NJ trees.

### Vector Construction and Rice Transformation

To construct the *RGG1*-OE vector, the full-length coding sequence was amplified from NIP cDNA and then inserted into the p1301Ubi vector. To generate pC1300-Cas9-g^*RGG1*^ mutants, we designed a target sequence in the first exon, and the final fragment was inserted into the pC1300-Cas9 vector. The 2.0-kb promoter sequence of *RGG1* was cloned to drive the β-glucuronidase (GUS) gene, and the promoter-GUS vector was transformed into NIP. All these vectors were introduced into *Agrobacterium tumefaciens* strain EHA105 for subsequent transformation of NIP or WYJ30. Homozygous T_2_ generation plants were used for further analysis.

All primers used in this study are listed in Supplemental Table S3.

### Histochemical GUS Staining and Subcellular Localization Analysis

For GUS staining, positive transgenic plants were selected at different developmental stages using an X-Gluc kit (Real-Times (Beijing) Biotechnology Co. Ltd.). Leaves, nodes, sheaths, stems, roots, and young panicles of different stages were collected and then soaked in solution with X-Gluc in a 37 °C dark environment for one night. Then, samples were cleared with absolute ethanol for observation.

For subcellular localization analysis, the full-length cDNA of the *RGG1* gene was amplified and cloned into the pCAMBIA1300-221GFP vector to generate 35S::RGG1-GFP. The construct was directly transformed into rice protoplasts, and the GFP signals were observed by confocal microscopy (Leica). The primers used are listed in Supplemental Table S3.

### Evaluation of Agronomic Traits

Before harvest, several yield-related agronomic traits were measured, including plant height, internode length, TN, and PL of the main stem. Grain-related traits, including grain length, grain width, and TGW, were measured after harvesting and stored at 37 °C for 1 week. The total seeds of one plant, minus any empty grains, were weighed to determine the grain yield per plant. Data statistics and sample *t-*tests were analysed using Excel (2016) software.

### Histological Analysis

Fresh young spikelet hulls of WYJ30 and the WYJ30-OE lines were collected, fixed in 2.5% glutaraldehyde for more than 24 h and then dehydrated through a graded alcohol-isoamyl acetate series. Images of cross-sections were taken on a Zeiss Axioskop HBO 50 or a Leica MZFLIII fluorescence stereomicroscope. For glume cell observation, the outer surfaces of mature seeds were observed by SEM (S-4800, Hitachi). The cell number and cell area in the outer parenchyma cell layer were measured using ImageJ software.

### RNA Extraction and qPCR

Total RNA was extracted using an RNA extraction kit (Beijing Tiangen Biotechnology Co. Ltd.). High-quality RNA was used to generate cDNA using a FastQuant RT Kit (Beijing Tiangen Biotechnology Co. Ltd.). Gene expression levels were analysed using qPCR. The rice *Actin* gene was used as an internal control. The qPCR was carried out in a total volume of 20 μL, containing 2 μL of cDNA, 10 μM of each primer, 10 μL of 2× SYBR Green PCR Master Mix, and 0.4 μL of 50 × ROX Reference Dye 2 (Vazyme Biotech Co. Ltd.), and performed on an ABI ViiA 7 Real-Time PCR System. The primers used for qPCR are listed in Supplemental Table S3.

### Cytokinin Measurement and Treatment

For measurement of cytokinin, young panicles of NIP and transgenic plants were collected in liquid nitrogen. Cytokinin measurement was performed as previously described (Cai et al. 2014). For cytokinin treatment, one-week-old seedlings were grown in hydroponic medium and then treated with different concentrations of 6-BA. After 1 week of treatment, the shoot and root length were measured to analyse the response to cytokinin. For gene expression analysis, 10-d-old seedlings were grown in hydroponic medium containing 10 μM 6-BA. Leaves were collected every 2 h for RNA extraction, and the expression level of *OsRR9* was detected by qPCR.

### RNA-Sequencing Analysis

Young panicles of NIP and the transgenic lines were collected for total RNA extraction using a TRIzol reagent kit (Invitrogen, Carlsbad, CA, USA). Construction of the cDNA library and sequencing were performed at Gene Denovo Biotechnology Co. (Guangzhou, China) using the Illumina HiSeq 2500 platform (Illumina Inc., San Diego, CA, USA). The filtered clean reads were aligned to the rice NIP reference genome and genes (http://rice.plantbiology.msu.edu/) using HISAT2. 2.4. RNA differential expression analysis was performed by DESeq2.

### Yeast Two-Hybrid Assay

To detect the interactions between RGG1 and RGB1, the full-length and truncated sequences of *RGG1* were cloned into the pGADT7 vector, and RGB1 was cloned into the pGBKT7 vector. Yeast two-hybrid assays were performed according to the manufacturer’s user manual. The primers listed in Supplemental Table S3.

### BiFC Analysis

For the BiFC assay, the coding sequence of RGG1 was cloned into pCAMBIA1300-35S-N-YFPn, and the coding sequence of RGB1 was cloned into the pCAMBIA1300-35S-N-YFPc vector. The plasmids were electroporated into *A. tumefaciens* (strain GV3101) and coinfiltrated into tobacco (*Nicotiana benthamiana*) leaves. After infiltration for 2–3 days, the GFP signals were observed by confocal microscopy (Leica). The primers used for BiFC are listed in Supplemental Table S3.

### Statistical Analysis

The results are presented as the mean ± SD. Microsoft Excel 2016 was used for statistical testing. GraphPad Prism 8 was used to produce bar charts and line charts. Significance levels were determined according to Student’s *t*-test: *P < 0.05, **P < 0.01.

## Supplementary Information

**Additional file 1 **: **Fig. S1.** Sequence alignment of the Gγ proteins in rice, Arabidopsis and maize. The red line indicates the predicted nuclear localization signal sequence. The red box indicates the CaaX isoprenylation motif at the C-terminal end. The green line indicates the GGL domain. **Fig. S2.** Subcellular localization of RGG1^ΔNLS^-GFP. Scale bar, 20 μm. **Fig. S3.** Internode lengths of NIP and the *RGG1* OE lines. Scale bar, 5 cm. **Fig. S4.** Comparison of plant and grain phenotypes between WYJ30 and the OE lines. **Fig. S5.** Targeted mutagenesis of RGG1 in the WYJ30 background and protein sequence alignment of WYJ30 and the mutants. **Fig. S6.** Gene Ontology (GO) DEG enrichment of the biological process, molecular function and cellular component categories. **Fig. S7.** Relative expression levels of genes concerning cytokinin biosynthesis. **Fig. S8.** NaCl treatment of NIP and the transgenic lines. **Table S1.** Comparison of major agronomic traits between NIP and the transgenic lines. **Table S2.** Comparison of major agronomic traits between WYJ30 and the transgenic lines. **Table S3.** Primers used in this study.

## Data Availability

The datasets supporting the conclusions of this article are included within the article and its additional files.

## Abbreviations

G proteins: Heterotrimeric GTP binding proteins
GUS: β-glucuronidase
NIP: Nipponbare
WYJ30: Wuyunjing 30
DEGs: Differentially expressed genes
6-BA: 6-benzylaminopurine
GPCRs: Seven-pass transmembrane G protein–coupled receptors
SAM: Shoot apical meristem
LOG: LONELY GUY
CT2: COMPACT PLANT2
CLV2: FASCIATE EAR2
BG3: Big Grain3
qPE9–1/DEP1: DENSE AND ERECT PANICLE 1
ren1-D: Root enhancer1
OsCKX4: Cytokinin oxidase 4
OsPUP4: Purine permease 4
GGL: G gamma-like
NLS: Nuclear location signal
BiFC: Bimolecular fluorescent complementation
qPCR: Quantitative reverse transcription-PCR
GFP: Green fluorescent protein
PL: Panicle length
TN: Tiller number per plant
GN: Grain number
TGW: 1000-grain weight
GL: Grain length
GW: Grain width
GYPP: Grain yield per plant
SEM: Scanning electron microscopy
KEGG: Kyoto Encyclopedia of Genes and Genomes
iP: Isopentenyladenine
tZ: Trans-zeatin
iPR: N6-(Δ2-isopentenyl) adenosine
tZR: Trans-zeatin-riboside
XLGs: EXTRA-LARGE Gα-like PROTEINs
